# Joining Alumina and Sapphire by Growing Aluminium Borate Whiskers In-Situ, and the Whiskers’ Orientation Relationship with the Sapphire Substrate

**DOI:** 10.3390/ma13010175

**Published:** 2020-01-01

**Authors:** Chun Li, Xiaoqing Si, Shuang Wu, Junlei Qi, Yongxian Huang, Jicai Feng, Jian Cao

**Affiliations:** State Key Laboratory of Advanced Welding and Joining, Harbin Institute of Technology, Harbin 150001, China; chun.li@hit.edu.cn (C.L.); sixq@hit.edu.cn (X.S.); 18801203918@163.com (S.W.); jlqi@hit.edu.cn (J.Q.); yxhuang@hit.edu.cn (Y.H.); feng_jicai@163.com (J.F.)

**Keywords:** alumina, alumina borate whiskers, texture, XRD

## Abstract

Bonding between polycrystal alumina and sapphire with (0001), (10 $\bar{1}$0), (11 $\bar{2}$0), (1 $\bar{1}$02) orientations is successfully achieved by growing aluminium borate whiskers in the joint. The morphology of the whiskers in the joint is characterised by (Scanning Electron Microscopy) SEM. The relationship between the growing direction of the aluminium borate whiskers and the orientation of the sapphire substrate is investigated. The effect of the growing direction of the aluminium borate whiskers on the mechanical properties of the joint is discussed. The results show that the whiskers on the sapphire with (10 $\bar{1}$0) orientation grow perpendicular to the surface of the substrate while the whiskers show a random growth on the other substrates. It is found that there is an orientation relationship between the whiskers (220) and sapphire (10 $\bar{1}$0) and the morphology of the whiskers has great influence on the mechanical properties of the joint. The joint between polycrystal alumina and sapphire with (10 $\bar{1}$0) orientation exhibits the highest strength, which reaches 26 MPa.

## 1. Introduction

Alumina is a kind of ceramic with high strength, outstanding electrical [1] and high temperature resistance [2], and excellent biocompatibility [3], which is a promising candidate for hip replacement material [4], abrasion protection material [5] and insulating barrier material in capacitors [6]. To realise these applications, alumina usually needs to be fabricated into complex structures. However, due to the high hardness and brittleness of the ceramic, it is very difficult to machine the ceramics. Manufacturing the complex structure by joining small pieces of ceramics with relatively simple shape together can be a reliable solution. Currently, the most commonly used method to join ceramic is by active metal brazing [7,8], which incorporates active elements such as Ti, Zr and Hf into the brazing filler to promote its reaction and wetting with alumina. However, due to the difference in the coefficient of thermal expansion (CTE) between alumina (8.3 × 10^−6^ K^−1^) [9] and filler metal (18.2 × 10^−6^ K^−1^ for the most commonly used AgCuTi brazing filler) [10], a significant level of residual stress can be generated during cooling of the brazing process [11,12]. The residual stress is detrimental to the reliability of the brazing joint and sometimes may lead to the direct failure of the joint during cooling. Also, the filler metal usually has much lower melting temperature than the ceramic which inevitably decreases the high temperature resistance of the manufactured structure [13]. 

Recently, a novel method was developed to join alumina by in-situ growing aluminium borate whiskers between the two substrates [14,15]. Since the difference in CTE between the whiskers (4 × 10^−6^ K^−1^) [16] and alumina is smaller than that between the metal brazing alloy and alumina, the residual stress in the joint can be reduced and the reliability of the joint can thus be improved. Lo et al. [14] realised the bonding of alumina by growing aluminium borate whiskers between the substrates and the bending strength of the joint reached 155 MPa. Cao et al. [15] also achieved the joining of alumina by synthesising aluminium borate whiskers and nanowire in the joint and the bending strength of the joint reached 92.8 MPa. Aluminium borate whiskers have outstanding high temperature resistance. Wang et al. [16] applied aluminium borate whiskers to join alumina and the strength of the achieved joint at 800 °C reached 27 MPa. It can be seen that joining alumina by growing aluminium borate whiskers in the joint is a promising way to reduce the residual stress and retain the high temperature strength of the alumina.

Since the whiskers are in-situ grown on the alumina substrate, the orientation of the whiskers could be affected by the orientation of the grain on which they grow. Some research has been carried out to investigate the relationship between the orientation of the whiskers and that of the semiconductor material substrate. Yazawa et al. [17] applied metalorganic vapour phase epitaxy (MOVPE) to grow InAs whiskers on GaAs substrate and found that the InAs whiskers preferred to grow parallel to the <111> direction of the substrate. Seifert et al. [18] also observed similar phenomenon and overcame the randomness in whisker growth. Up to now, there is little investigation on the orientation relationship between the whiskers and the substrate for ceramic joining. The only available report is that Wang et al. [16] which investigated on the relationship between orientation of the whisker and that of the grain where it grows. It was found that there was a semi-coherent relationship between the (220) planes of Al_4_B_2_O_9_ and the (100) planes of the Al_2_O_3_ substrate. However, the authors applied TEM to observe only one interface between a whisker and the grain it grows on. This result cannot show the statistic orientation relationship between the whiskers and the substrate. Also, the authors used polycrystal alumina as the substrate, the grain orientations of which are random. This makes it difficult to investigate the general relationship between the orientation of the whiskers and the alumina grains it grows on. Sapphire is single crystal alumina with uniform orientation on its surface. The orientation relationship between the whiskers and the substrate can thus be studied by using sapphire as the substrate. 

Aluminium borate whiskers are single crystals and the mechanical properties of which are highly anisotropic. Thus the properties of the joint can be greatly influenced by the growing direction of the whiskers in the joint. However, no research has been carried out to investigate the effect of the whisker growing direction on the mechanical properties of the joint.

Thus in this paper, we achieved the joining between sapphire with four different orientations and polycrystal alumina by growing aluminium borate whiskers in the joint. The growing directions of the whiskers grown on sapphire with various surface orientations and polycrystal alumina are observed by SEM. The relationship between the orientation of the whiskers and the substrates they grow on is characterised by (X-ray diffraction) XRD using a texture analysis approach. The effect of the growing direction of the whiskers on the mechanical properties of the joint is studied.

## 2. Materials and Methods 

The sapphire substrates with the size of 10 mm × 10 mm × 0.5 mm were manufactured by HeFei Crystal Technical Material Co.,Ltd, (Hefei, China). Four types of substrates which have (0001), (10 $\bar{1}$0), (11 $\bar{2}$0), (1 $\bar{1}$02) orientations are applied. The polycrystal alumina was cut into 5 mm × 5 mm × 10 mm pieces by the Diamond cutting wheel. Prior to joining, the substrates were ultrasonically cleaned in acetone for 10 min. The interlayer utilized for joining is B_2_O_3_ powder which is supplied by Shanghai Unite Technology Co., Ltd, (Shanghai, China). The interlayer was placed between the two substrates and a small load of 5 kPa was applied on the top surface of the assembly to ensure the contact between the substrate and the interlayer and to squeeze out the excess liquid. The joining process was carried out in a muffle furnace at 950 °C for 6 h. At this temperature, the following reaction between the interlayer and the substrate will be carried out [14,19].

(1) $$2Al_{2}O_{3}+B_{2}O_{3}\to 2Al_{2}O_{3}\cdot B_{2}O_{3}$$

**Figure 1:**
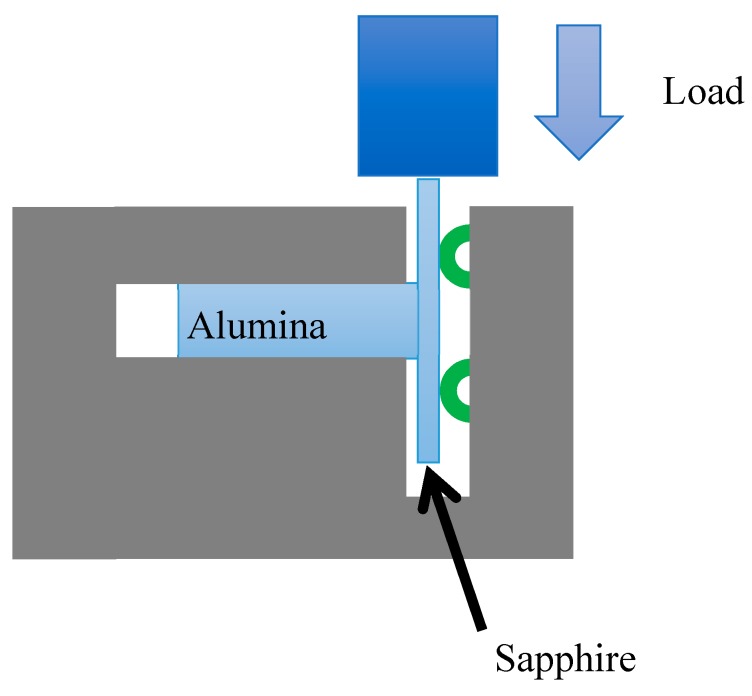
The schematic of the shear test for the joint, where the polycrystal alumina is fixed by the rig and a load is applied on the sapphire by the universal mechanical testing machine to generate a shear stress at the interface.

The substrates will be bonded by the grown 2Al_2_O_3_ B_2_O_3_ whiskers. After the joining process, the samples were carefully cross sectioned, ground and polished to 1 μm. A thin layer of gold coating (~20 nm thick) was applied on the polished surface of the sample and the microstructure of the joint was observed by SEM (Quanta 200, Thermo Fisher Scientific, Hillsboro, OR, USA) coupled with an EDS detector (QUANTAX, Bruker, Berlin, Germany). The mechanical property of the joint was characterised by shear test and the schematic of the test is shown in Figure 1. 

As shown in Figure 1, during testing, the polycrystalline alumina was fixed and the load was applied on the sapphire with a speed of 0.5 mm/min. To investigate the relationship between the orientation of the whiskers and that of the substrate, the texture of the whiskers on the fracture surface of the joint at the sapphire side was investigated by XRD (X’pert MRD Panalytical, Almelo, The Netherlands) with a copper K_α_ tube and an Euler cradle. The pole figure was measured to determine the texture of the aluminium borate whiskers grown on the various sapphire substrates. The measured results was analysed using the X’pert texture analysis software (Panalytical, Almelo, The Netherlands). 

## 3. Results and Discussion

**Figure 2:**
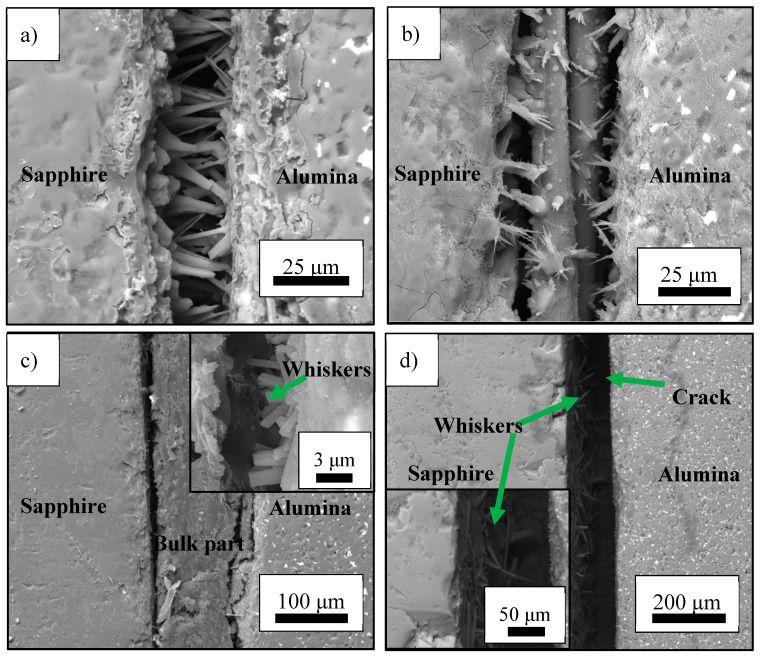
The microstructure of joint between polycrystal alumina and sapphires with various orientations, (**a**) The joint between polycrystal alumina and sapphire with (10 $\bar{1}$0) orientation where the whiskers grow vertically to the surface of the sapphire, (**b**) The joint between polycrystal alumina and sapphire with (11 $\bar{2}$0) orientation, (**c**) The joint between polycrystal alumina and sapphire with (0001) orientation, the insert shows the magnified image of the interfacial microstructure on alumina side, (**d**) The joint between polycrystal alumina and sapphire with (1 $\bar{1}$02) orientation, the insert shows the magnified image of the interfacial microstructure on sapphire side.

The typical microstructure of the joint between sapphire with different orientations and polycrystal alumina is shown in Figure 2. 

**Figure 3:**
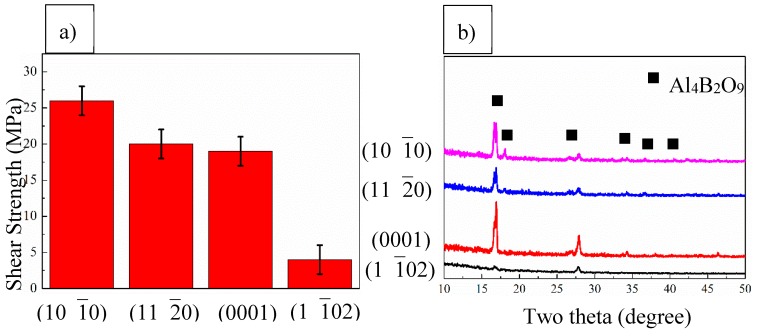
(**a**) The shear strength of the joint between polycrsystal alumina and sapphire with different orientations, (**b**) The XRD (X-ray diffraction) pattern of the fracture surface on the sapphire side, where Al_4_B_2_O_9_ whiskers can be indexed and the intensity of the peaks differs from each other.

It can be seen that polycrystal alumina is successfully joined with sapphire by in-situ growing whiskers between the two substrates. However, the morphology of the whiskers in the joint could vary a lot with the orientation of the sapphire substrates. As shown in Figure 2a, when using the sapphire with (10 $\bar{1}$0) orientation as the substrate, the whiskers are well aligned and grow almost perpendicular to the surface of sapphire. The diameter of the aluminium borate whiskers are generally uniform and most of the whiskers could grow through the joint, connecting the two substrates. Two kinds bonding mechanism could contribute to this type of joint: the first mechanism is the joining achieved by the whiskers that grow through the joint and the second one is the mechanical knitting between the whiskers grown from the sapphire and the polycrystal alumina. This column structure of the seam could help to increase the strain compliance of the joint, which is beneficial to the reliability of the joint. The microstructure of the joint between the polycrystal alumina and the sapphire with (11 $\bar{2}$0) orientation is presented in Figure 2b. As can be seen in the figure, the joint mainly consists of three parts: the whiskers grown on the surface of sapphire, a bulk part in the middle which may be the unreacted B_2_O_3_ and the whiskers on the polycrystal alumina. Both the density and the length of the aluminium borate whiskers on sapphire with (11 $\bar{2}$0) orientation is much lower than that of the whiskers grown on the sapphire with (10 $\bar{1}$0) orientation. The whiskers on the polycrystal alumina also show a random growth and the length of which is shorter on the than the whiskers on the sapphire side. Since in this case, the joining between the two substrates is realised by the mechanical knitting between the whiskers, which may not be as reliable as the joint achieved by using sapphire with (10 $\bar{1}$0) orientation as the substrate. The typical microstructure of the joint between the sapphire with (0001) orientation and the polycrystal alumina is demonstrated in Figure 2c. Two main parts can be observed in the joint: the first one is a bulk part which could be the unreacted B_2_O_3_, while the second part is the whiskers on the polycrystal alumina. It is noted that no aluminium borate whiskers can be found on the surface of the sapphire with (0001) orientation. This may be due to the incompatible between the preferred whisker growth direction and the orientation of the substrate. The morphology of the aluminium borate whiskers on the surface of the polycrystal alumina substrate is similar to the other joints, which has a relatively short length and random growth directions. For this type of joint, the main part in the joint is the bulk brittle material, the deformation ability of which is quite limited. Due to the absence of the column structured whiskers, the strain compliance of the joint is decreased, which may be detrimental to the reliability of the joint. Figure 2d depicted the microstructure of the joint between the sapphire with (1 $\bar{1}$02) orientation and the polycrystal alumina. Large cracks can be observed in the middle of the joint. Aluminum borate whiskers can be observed on both the sapphire and alumina substrate. Most of the whiskers on the sapphire substrate “lay down” on the surface and the morphology of the whiskers on alumina is similar to the other joints. No connections between the whiskers from both substrates can be found, which may lead to relatively poor joining quality. From the above discussion, it can be seen that the growing direction of the aluminium borate whiskers could be affected by the orientation of the substrate it grows on. Since aluminium borate whiskers are single crystal materials with anisotropic mechanical properties, the strength of the joint will be influenced by the morphology of the whiskers in the seam. The mechanical properties of the joint were characterised by shear test and the result is shown in Figure 3a.

**Figure 4:**
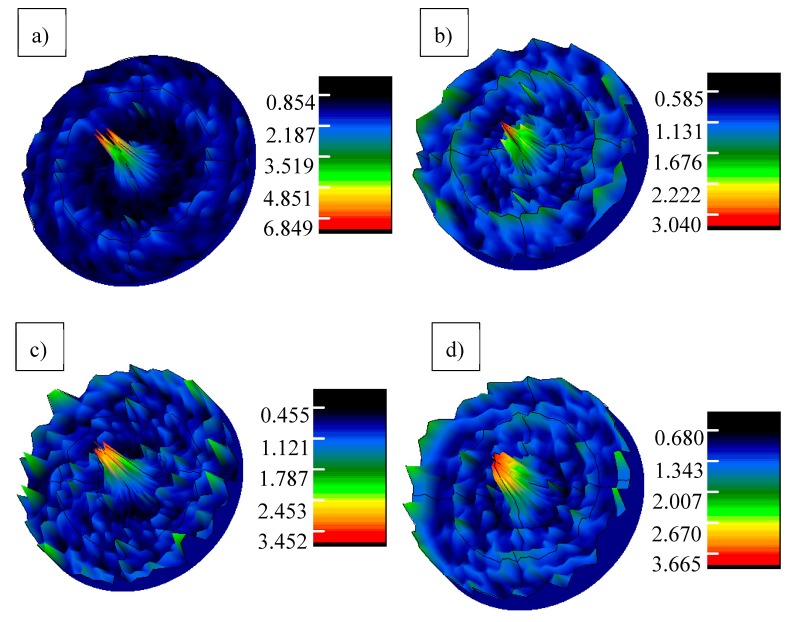
The {220} pole figure of the aluminium borate whiskers grown on various substrates, (**a**) The {220} pole figure of the aluminium borate whiskers grown on sapphire with (10 $\bar{1}$0) orientation, (**b**) The {220} pole figure of the aluminium borate whiskers grown on sapphire with (11 $\bar{2}$0) orientation, (**c**) The {220} pole figure of the aluminium borate whiskers grown on sapphire with (0001) orientation, (**d**) The {220} pole figure of the aluminium borate whiskers grown on sapphire with (1 $\bar{1}$02) orientation.

From Figure 3a, we can see that the joint between the sapphire with (10 $\bar{1}$0) orientation and polycrystal alumina has the highest shear strength of ~26 MPa; When using the sapphire with (11 $\bar{2}$0) and (0001) orientations as the substrates, the shear strength of the joint drops slightly to around 20 MPa and the strength of the joint between the sapphire with (1 $\bar{1}$02) orientation and polycrystal alumina shows the lowest shear strength of about 4 MPa. When using the sapphire with (10 $\bar{1}$0) orientation as the substrate, the seam processes a column microstructure, which offers the joint strain compliance and result in the high shear strength of the joint. However, for the joint with (11 $\bar{2}$0) and (0001) orientations as the substrates, even though the joints are still well bonded, bulk microstructure appears in the joint. These bulk parts are made of brittle phases which have little ability to deform, decreasing the strain compliance and the strength of the joint. For the joint between the sapphire with (1 $\bar{1}$02) orientation and polycrystal alumina, large cracks exist in the joint and no connection can be found between the whiskers on the two substrates. Thus the strength of the joint is relatively low. After the shear test, the fracture of the joint on the sapphire side was characterised by XRD with a grazing angle scanning mode, the incident angle of which was fixed at 3° and the scanning range is from 10° to 50°. The achieved patterns are shown in Figure 3b. It can be seen that the main phases in the joint are Al_4_B_2_O_9_ whiskers. It needs to be addressed that the intensity of the achieved of the patterns from the whiskers on various substrates are different from each other and also different from the standard Al_4_B_2_O_9_ whisker pattern. For the whiskers grown on sapphire with (11 $\bar{2}$0), (0001) and (1 $\bar{1}$02) orientations, only (220) and (231) peaks can be observed, while for the whiskers grown on sapphire with (10 $\bar{1}$0) orientation, the (111) peak can also be indexed in the pattern. The intensity of the (231) peak in XRD pattern of the whiskers grown on sapphire with (0001) orientation is larger than that of the (220) peak and for the other three patterns, the (220) peak intensity is higher. This may be due to the texture of the grown whiskers. Thus to analyse the texture of the aluminium borate whiskers and investigate the relationship between the growing direction of the whiskers and the orientation of the substrate, the XRD texture analysis approach was carried out. The archived patterns are firstly imported into the X’Pert texture analysis software to carry out the background correction, then the (orientation distribution function) ODF is calculated from the corrected data and the pole figures are achieved from the calculated ODF. The pole figures are depicted in Figure 4. 

As shown in Figure 4a, the {220} pole figure presents a predominance of the (220) planes parallel to the surface of the sapphire with (10 $\bar{1}$0) orientation. This result corresponds well with the (transmission electron microscopy) TEM observation result of Wang et al. [16]. For the whiskers grown on sapphire with (11 $\bar{2}$0), (0001) and (1 $\bar{1}$02) orientations, the intensity of the pole figures is uniformly distributed and the highest intensity is relatively low (around 3), which indicates that there is no strong texture for the whiskers grown on these three kinds of substrates and thus no obvious correlation between the whiskers growing direction and the orientation of the substrate. This correlation could give some indication for controlling the growing direction of the aluminium borate whiskers on sapphire substrate.

## 4. Conclusions

In summary, we achieved the joining between polycrystal alumina and sapphire with various orientations by in-situ growing aluminium borate whiskers between the two substrates. It was found that the growing direction of aluminium borate whiskers is related to the orientation of the substrate and the aluminium borate whiskers on the surface of sapphire with (10 $\bar{1}$0) orientation grow vertically to the substrate and have a uniform distribution. The aluminium borate whiskers on the surface of sapphire with (11 $\bar{2}$0), (0001) and (1 $\bar{1}$02) orientations show a random growth. The morphology of the aluminium borate whiskers could influence the reliability of the joint and the joint between polycrystal alumina and the sapphire with (10 $\bar{1}$0) orientation is found to exhibit the highest shear strength and reached 26 MPa.
